# CRISPR interference: a structural perspective

**DOI:** 10.1042/BJ20130316

**Published:** 2013-06-28

**Authors:** Judith Reeks, James H. Naismith, Malcolm F. White

**Affiliations:** Biomedical Sciences Research Complex, University of St Andrews, St Andrews, Fife KY16 9ST, U.K.

**Keywords:** antiviral defence, cluster of regularly interspaced palindromic repeats (CRISPR), crystallography, evolution, protein structure, repeat-associated mysterious protein (RAMP), BhCas5c, *Bacillus halodurans* Cas5c, CRISPR, cluster of regularly interspaced palindromic repeats, Cas, CRISPR-associated, Cascade, CRISPR-associated complex for antiviral defence, crRNA, CRISPR RNA, dsDNA, double-stranded DNA, EcoCas3, *Escherichia coli* Cas3, EM, electron microscopy, HD, histidine–aspartate, MjaCas3″, *Methanocaldococcus jannaschii* Cas3″, PaCas6f, *Pseudomonas aeruginosa* Cas6f, PAM, protospacer adjacent motif, PfuCas, *Pyrococcus furiosus* Cas, pre-crRNA, precursor crRNA, RAMP, repeat-associated mysterious protein, RRM, RNA recognition motif, ssDNA, single-stranded DNA, SsoCas, *Sulfolobus solfataricus* Cas, ssRNA, single-stranded RNA, SthCas3, *Streptococcus thermophilus* Cas3, tracrRNA, *trans*-activating crRNA, TtCas, *Thermus thermophilus* Cas

## Abstract

CRISPR (cluster of regularly interspaced palindromic repeats) is a prokaryotic adaptive defence system, providing immunity against mobile genetic elements such as viruses. Genomically encoded crRNA (CRISPR RNA) is used by Cas (CRISPR-associated) proteins to target and subsequently degrade nucleic acids of invading entities in a sequence-dependent manner. The process is known as ‘interference’. In the present review we cover recent progress on the structural biology of the CRISPR/Cas system, focusing on the Cas proteins and complexes that catalyse crRNA biogenesis and interference. Structural studies have helped in the elucidation of key mechanisms, including the recognition and cleavage of crRNA by the Cas6 and Cas5 proteins, where remarkable diversity at the level of both substrate recognition and catalysis has become apparent. The RNA-binding RAMP (repeat-associated mysterious protein) domain is present in the Cas5, Cas6, Cas7 and Cmr3 protein families and RAMP-like domains are found in Cas2 and Cas10. Structural analysis has also revealed an evolutionary link between the small subunits of the type I and type III-B interference complexes. Future studies of the interference complexes and their constituent components will transform our understanding of the system.

## INTRODUCTION

CRISPRs (cluster of regularly interspaced palindromic repeats) are a prokaryotic defence mechanism against viral infection and horizontal gene transfer. CRISPRs are the largest family of prokaryotic repeats [1] and have been found in 48% of bacterial and 84% of archaeal sequenced genomes to date [2]. A CRISPR array consists of a series of short identical repeat sequences separated by similarly short variable sequences known as spacers [3]. Located adjacent to the CRISPR array are clusters of *cas* (CRISPR-associated) genes [4] that encode for the proteins responsible for mediating the CRISPR response to foreign nucleic acids. The spacers are derived from foreign nucleic acids, such as viruses and conjugative plasmids, and provide the host with a ‘genetic memory’ of threats previously encountered [1,5,6]. New spacers are captured in a poorly understood process known as ‘adaptation’ and incorporated into the CRISPR locus [7]. The spacers are used to target foreign nucleic acids containing sequences complementary to the spacer, termed protospacers, for degradation [8]; the process is termed ‘interference’.

**Figure 1:**
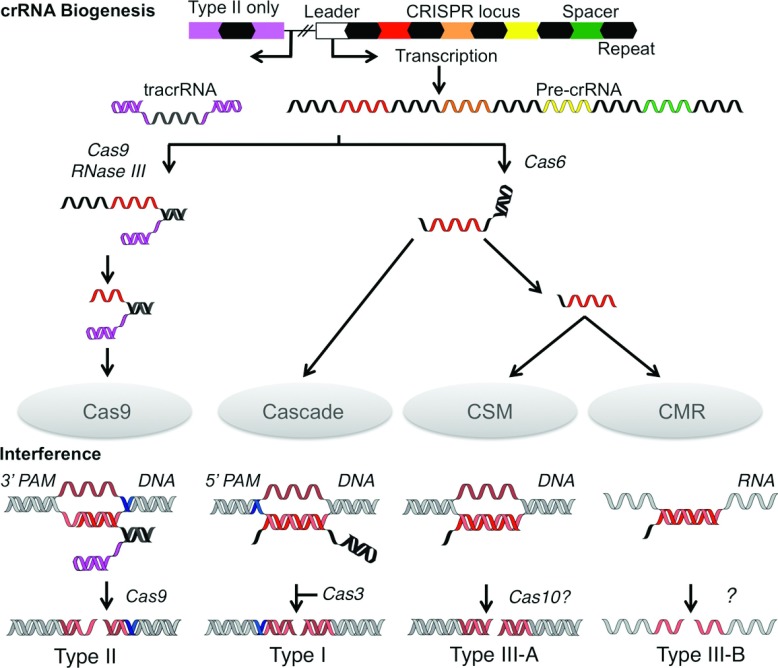
Schematic representation of crRNA biogenesis and CRISPR interference Processing events involving nucleic acids are coloured; repeats (black), spacers (red–green) and tracrRNA (magenta). For clarity, a single spacer (red) was used to illustrate the processes, although in actual systems all spacers are processed. Targets are shown in other red shades (lighter for the complementary strand and darker for the non-complementary). The PAMs are shown in blue. The pre-crRNA and interference nucleases are indicated along with the interference complexes.

The first step in the interference pathway is the transcription of the CRISPR array from a promoter located in the ‘leader’ sequence, an AT-rich region located upstream of the CRISPR array [4,9]. The array transcript {pre-crRNA [precursor crRNA (CRISPR RNA)]} is then processed into short crRNAs containing a spacer and flanking repeat fragments (Figure 1) [10]. These crRNAs are subsequently bound by complexes of Cas proteins and used to target homologous foreign dsDNA (double-stranded DNA) or ssRNA (single-stranded RNA) for nucleolytic degradation during CRISPR interference (Figure 1) [8,11].

**Figure 2:**
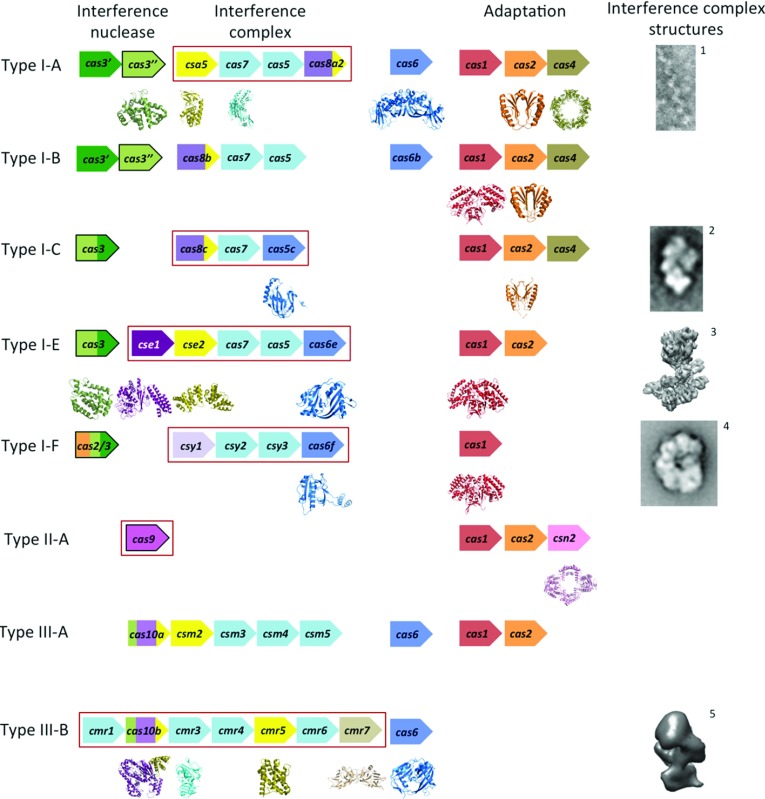
The CRISPR/Cas systems and their respective proteins Typical gene identities are shown for CRISPR/Cas subtypes according to the recent classification by Makarova et al. [12]. The genes are ordered by function: interference (left) and adaptation (right). The interference proteins are subdivided into the interference nuclease (left, outlined in black), proteins of the interference complex (middle, boxed in red) and pre-crRNA nucleases (right, although some are integral subunits of the interference complexes). The genes are coloured according to conserved domain and protein folds: catalytic RAMPs are shown in blue, non-catalytic RAMPs in light blue, HD nuclease domains in light green, Cas3 helicase domains in dark green, the large subunits in various shades of purple and the small subunits in yellow. Subtypes I-D and II-B are not shown as there is no directly relevant structural data. EM images and structures of the interference complexes (or subcomplex for I-A) are adapted from references ^1^ [13], ^2^ [14], ^3^ EMD-5314, ^4^ [15] and ^5^ [20].

The CRISPR/Cas systems are divided into three main types (I, II and III) on the basis of the identity and organisation of genes within a *cas* locus [12]. These types are further divided into a total of ten subtypes (I-A, I-B and so on), each of which expresses a different protein complex responsible for interference (Figures 1 and 2). The Cascade (CRISPR-associated complex for antiviral defence) is the effector complex for type I systems [8,13–15]. This name was originally used solely for the type I-E complex [8], which we here call *e*Cascade, but increasingly Cascade is used more as a general term for all type I complexes. Type II systems use a single protein for interference (Cas9) [16], whereas the III-B subtype uses the CMR complex [11]. The interference complex of the III-A subtype has yet to be characterized biochemically, but the similarity of the III-A and III-B operons suggests that interference is indeed mediated by an effector complex rather than a single protein. As a result the putative complex has been termed the CSM complex [12]. Every CRISPR/Cas system apart from the III-B subtype is thought to target dsDNA by forming an R-loop structure, consisting of a heteroduplex between crRNA and the complementary protospacer strand and a ssDNA (single-stranded DNA) non-complementary strand, followed by degradation by the interference nuclease (Figure 1) [8,17–19]. The CMR complex targets ssRNA by forming an RNA duplex, which is subsequently cleaved [11,20].

The mechanisms of adaptation and CRISPR interference have been extensively reviewed (see references [21–26]). In the present review we will focus on the structural biology of the CRISPR system. Crystal structures are available for eight of the ‘core’ Cas proteins (those found in multiple subtypes) as well as a number of subtype-specific proteins (Figure 2 and Supplementary Table S1 at http://www.biochemj.org/bj/453/bj4530155add.htm). The structures of proteins involved in spacer acquisition have provided interesting insights into their function within the CRISPR/Cas system as well as to similarities to non-Cas proteins, such as the parallels between Cas2 and VapD of the toxin/antitoxin system [27], but will not be discussed further in the present review. EM (electron microscopy) images and structures have been determined for five interference complexes, providing invaluable information on the function of each subunit. CRISPR systems are remarkably diverse and subject to rapid evolutionary change. Analysis of the key structural features of Cas proteins involved in crRNA biogenesis and interference highlights recurring themes and points to evolutionary relationships between apparently distinct protein families.

## PRE-crRNA PROCESSING AND crRNA BIOGENESIS

crRNA provides the CRISPR/Cas system with the sequence specificity needed to selectively target foreign nucleic acids. Mature crRNAs are produced from a single long transcript of the CRISPR array (pre-crRNA), which is processed to yield spacers with 5′ and/or 3′ repeat fragments (Figure 1) [10,28,29]. The method and nature of pre-crRNA processing is dependent on the CRISPR/Cas system. Type I and III systems use the Cas6 endonuclease to cleave pre-crRNA within the repeat sequence [8,13,15], with the exception of I-C systems that instead use a catalytic variant of Cas5 [14,30]. The crRNAs from various type III systems are further processed to reduce or remove the repeat sequence at the 3′ end [11,31]. The enzyme responsible for this degradation is not yet known. The type II system uses a very different mechanism, requiring the transcript of an anti-sense near-perfect repeat and flanking sequences [tracrRNA (*trans*-activating crRNA)] located adjacent to the CRISPR array for processing [32]. The duplex formed by pre-crRNA and tracrRNA is bound by Cas9 and cleaved in the repeat sequence by cellular RNase III and then in the spacer by an unknown nuclease to leave a spacer fragment and a 3′ repeat fragment [32].

Cas6 and the catalytic type I-C Cas5 [Cas5c, also confusingly known as Cas5d (Dvulg subtype)] catalyse the same reaction. The pre-crRNA is cleaved upstream of the spacer (8 nt for Cas6, 11 nt for Cas5c) [8,13,14,33,34] generating crRNA with a 5′ repeat-derived sequence known as the ‘5′-handle’ or ‘5′-tag’ that is critical for interference [20,35]. CRISPR repeats can be divided into twelve families based on sequence and secondary structure [36]. Cas5c targets repeats containing hairpin structures, whereas the subfamilies of Cas6 proteins, which broadly align with the CRISPR/Cas system with which they are associated, can cleave either unstructured or hairpin-containing repeats [14,33,37,38].

In type I systems, Cas6 can form an integral part of Cascade or it can exhibit a more transient interaction. Cas6e and Cas6f remain tightly bound to their cleaved products with low or sub-nanomolar affinities, and form part of their respective Cascades [15,37,39,40]. In fact, the type I-F complex (*f*Cascade) assembles specifically around a pre-formed Cas6f/crRNA complex [41]. Cas6 interacts more transiently with the I-A archaeal Cascade (*a*Cascade) [13,42]. Cas6 is not part of the type III-B CMR complex [11,20], and the associations of Cas6 with the type I-B, I-D and III-A complexes are unclear.

### The structures of Cas5c and Cas6

**Figure 3:**
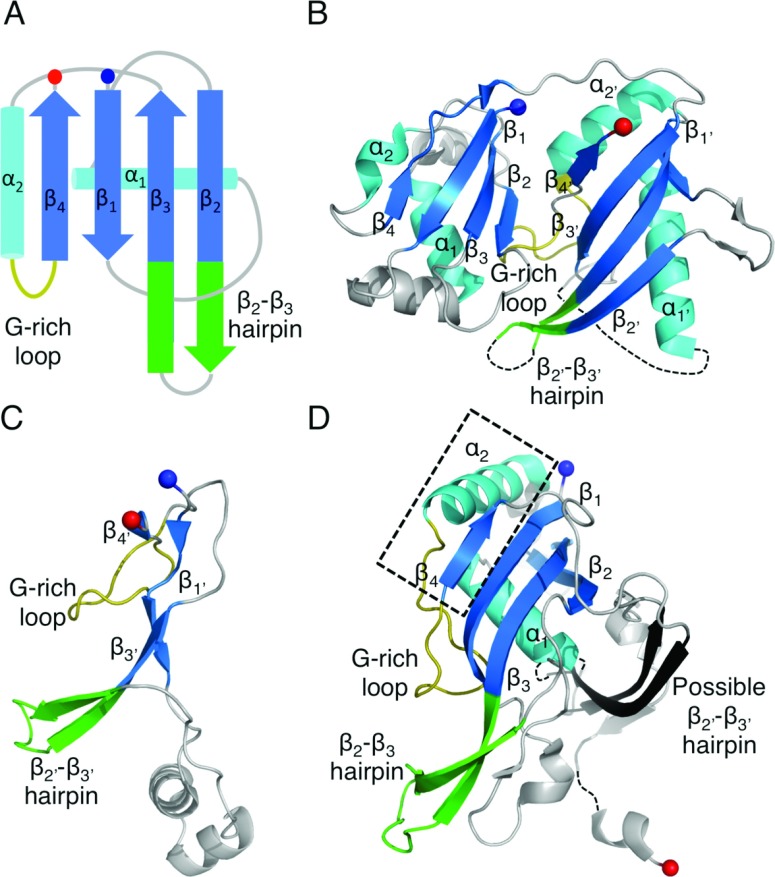
The structures of catalytic RAMP proteins (**A**) Topology diagram of a RAMP domain. The β-strands are shown in blue and the α-helices in cyan. The glycine-rich loop found in many RAMPs is shown in yellow and the β_2_–β_3_ hairpin observed in some RAMPs is shown in green. The N- and C-termini are shown as blue and red spheres respectively. (**B**) The structure of TtCas6e (PDB code 1WJ9) highlighting the two RAMP domains that may have arisen from a pseudo-duplication event. Secondary structural elements are labelled as described in the text. Conserved RAMP elements are coloured as in (**A**) and non-conserved elements in grey. Disordered regions are shown as broken black lines. (**C**) The atypical C-terminal domain of PaCas6f (PDB code 2XLK) that probably diverged from the standard RAMP fold. The recognizable features are labelled. (**D**) The structure of BhCas5c (PDB code 4F3M), a catalytic variant of the typically non-catalytic Cas5 family. The short β_4_ strand and parallel α_2_ helix are boxed in black. The possible β_2′_–β_3′_ hairpin in the C-terminal domain is shown in black.

Cas5 and Cas6 both belong to the RAMP (repeat-associated mysterious protein) superfamily. These proteins contain one or more RAMP domains, which form ferredoxin-like folds similar to that of the RRM (RNA recognition motif) domain [43], consisting of a four-stranded antiparallel β-sheet (arranged as β_4_β_1_β_3_β_2_) flanked on one face by two α-helices located after β_1_ and β_3_ in a βαββαβ fold (Figure 3A). Five conserved sequence motifs have been detected in the superfamily; as yet no single protein has been found to contain all five [44].

Cas6 proteins typically contain two sequential RAMP domains with the glycine-rich loop (motif V of the RAMP superfamily sequence motifs) located between α_2′_ and β_4′_ of the second (C-terminal) domain (the prime denotes a structural element in the second domain) (Figure 3B) [45–50]. This loop often fits the consensus sequence GΦGXXXXXGΦG, where Φ is a hydrophobic residue, X is any residue and the variable region contains at least one positively charged residue [51]. Other than this motif, the Cas6 proteins exhibit minimal sequence similarity. PaCas6f (*Pseudomonas aeruginosa* Cas6f) is atypical because it contains what is possibly a severely degraded C-terminal RAMP domain (Figure 3C) [33]. The C-terminal domain contains four short β-strands that, although they are orientated to form a RAMP β-sheet, are not aligned to do so (Figure 3C). The RAMP helices are not present, but the glycine-rich loop (albeit differing from the consensus sequence) is located between the correct β-strands. The Cas6 homologues contain additional secondary structure elements relative to the RAMP elements, but only one feature is fully conserved: a β-hairpin connecting β_2′_ and β_3′_ in the C-terminal domain (we denote this the β_2′_–β_3′_ hairpin) that extends beyond the β-sheet. This hairpin is even conserved in the abnormal C-terminal domain of PaCas6f.

Cas5c contains an N-terminal RAMP domain and a C-terminal domain consisting of a three-stranded antiparallel β-sheet (Figure 3D) [14,30,52]. The RAMP domain contains a glycine-rich loop that does not match the Cas6 consensus sequence. It also contains a β_2_–β_3_ hairpin that is joined by another short β-strand to form a β-sheet. In some Cas5c homologues, two helices are inserted into the tip of the hairpin [52]. Due to the hairpin and the glycine-rich loop, this RAMP domain is similar to the Cas6 C-terminal domain, although it also exhibits significant similarity to the N-terminal domain of archaeal Cas6 proteins. In Cas5c, α_2_ is not located behind β_4_; instead, the shorter β_4_ (in other RAMPs, β_4_ is longer or is followed by an extended strand) allows α_2_ to run antiparallel to β_1_ (compare Figure 3B with Figure 3D). This atypical arrangement could correctly position the residues of the active site, which is located at the intersection of α_1_ and α_2_ at the top of the β-sheet, a location different to that of Cas6 (see below). The β-sheet of the C-terminal domain does not have a RAMP domain arrangement of secondary structure elements. However, β_1′_ and β_2′_ form an extended β-hairpin reminiscent of the β_2′_–β_3′_ hairpin of Cas6, although this is the only feature that is potentially RAMP-like. Thus it is not possible to say with certainty whether the C-terminal domain of Cas5c is a highly divergent RAMP domain.

### RNA binding and cleavage

**Figure 4:**
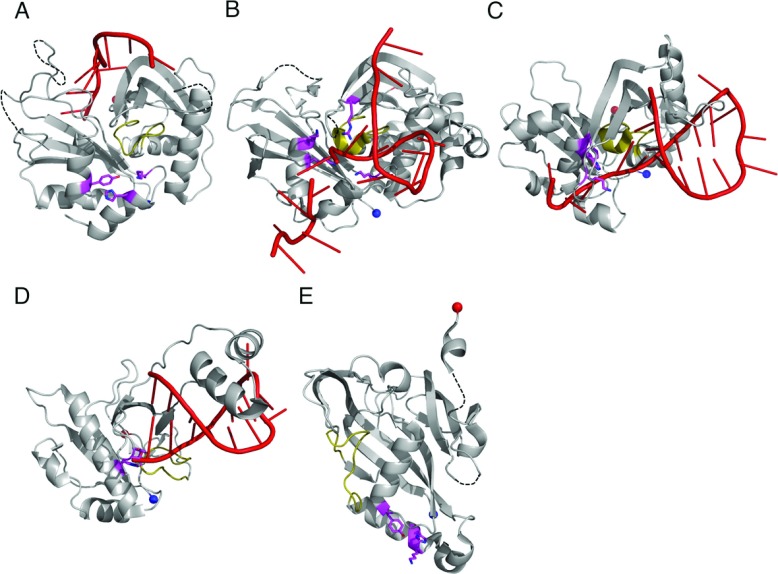
RNA binding and catalysis by Cas6 and Cas5c The structures of (**A**) PfuCas6 (PDB code 3PKM), (**B**) SsoCas6 (PDB code 4ILL), (**C**) TtCas6e (PDB code 2Y8W) and (**D**) PaCas6f (PDB code 2XLK) in complex with RNA (red). The glycine-rich loop is shown in yellow and the catalytic residues as magenta sticks. (**E**) The structure of BhCas5c (PDB 4F3M) highlighting the position of the active site (magenta). The four structures are shown to the same scale and same orientation. A three-dimensional representation of this Figure is available at http://www.biochemj.org/bj/453/0155/bj4530155add.htm.

Cas5c and Cas6 are both metal-independent ribonucleases that form products with 5′-hydroxyls and 2′,3′-cyclic phosphates [30,33,46,53], indicative of a general acid/base mechanism involving nucleophilic attack by the deprotonated 2′-hydroxyl on the scissile phosphate. The active site of Cas6 is located between α_1_ and the glycine-rich loop, although the exact position of the site varies amongst the subfamilies (Figures 4A–4D). Remarkably, the catalytic residues also vary between the proteins and none of the residues are conserved in all of the Cas6 subfamilies. Cas6 enzymes from *Pyrococcus furiosus* (PfuCas6) and *Thermus thermophilus* (TtCas6) possess a catalytic triad of histidine, tyrosine and lysine residues similar to the RNA-splicing endonuclease [37,46,54,55]. The tyrosine residue has been assigned as the general base and the histidine residue as the general acid, with the lysine residue stabilizing the pentacoordinate phosphate intermediate. PaCas6f, however, uses a catalytic dyad of histidine and serine residues, with the histidine residue acting as the general base and the serine residue holding the ribose ring in the correct conformation [41]. Two active Cas6 paralogues from *Sulfolobus solfataricus* contain neither a general acid nor a general base, instead using conserved positively charged residues to correctly orientate the substrate and stabilize the pentacoordinate phosphate intermediate [49,50]. The presence of a catalytic histidine residue in the N-terminal domain had previously been highlighted as a characteristic feature of Cas6s [56], but it is now clear that this is not necessarily the case.

The location of the Cas5c active site is different to that of Cas6, suggesting that the active sites evolved independently of each other. The catalytic triad of BhCas5c (*Bacillus halodurans* Cas5c) consists of a tyrosine residue located in α_1_ and histidine and lysine residues in α_2_, similar to the PfuCas6 and TtCas6e active sites [14]. The lysine is the only residue of the triad that is invariant across the family; the tyrosine residue can be exchanged for histidine (as in the active Cas5c nucleases from *Mannheimia succiniciproducens* and *Xanthomonas oryzae* [30,52]), phenylalanine or leucine, whereas the catalytic histidine residue can be replaced by other aromatic residues (phenylalanine/tyrosine) (Supplementary Figure S1 at http://www.biochemj.org/bj/453/bj4530155add.htm), but the roles of the residues are not yet understood. None of these supposed catalytic residues are conserved in other Cas5 proteins, perhaps unsurprisingly since only Cas5c is catalytically active.

As expected for nucleases that process RNA substrates with a range of secondary structures, multiple modes of RNA binding have been observed across the Cas6 family. This perhaps underlies the variation in the position of the active site as the different modes alter the position of the scissile bond. PfuCas6 and its inactive homologue from *Pyrococcus horikoshii* (PhCas6nc) bind unstructured RNA in a ‘wrap-around’ mechanism where the RNA binds in the cleft between the two domains (Figure 4A) [38,48]. These enzymes bind the 5′ end of the repeat in the cleft between the β-sheets of the two domains and this interaction with the first ~10 nt appears to be the predominant determinant of binding affinity. Although the 3′ end of the substrate, including the scissile phosphate, is disordered in the crystal structures, it is predicted to follow the positively charged cleft into the active site [38]. TtCas6e, PaCaf6f and a homologue from *S. solfataricus* (SsoCas6) bind hairpin RNA with the majority of the contacts formed by the C-terminal domain (Figures 4B–4D). TtCas6e and SsoCas6 bind the hairpin across the helical face of the protein using a series of basic residues to bind the phosphate backbone of the 3′ strand of the hairpin [37,49,55]. The RNA hairpin of SsoCas6 is shorter than that of TtCas6e by 3 bp and is predicted to be unstable in solution [36], meaning that SsoCas6 specifically stabilizes the hairpin conformation. PaCas6f, which shares few C-terminal secondary structure elements with other Cas6 proteins, binds the RNA hairpin between the RAMP β-strands and a helix–loop–helix motif, using the first helix to bind the major groove of the RNA [33]. In all three of these proteins, the β_2′_–β_3′_ hairpin is inserted into the base of the RNA hairpin, serving to position the scissile phosphate within the active site and, in the case of PaCas6f and SsoCas6, provides key catalytic residues. It seems likely that the β_2′_–β_3′_ hairpin plays a conserved role across the Cas6 family.

The method of substrate binding in Cas5c must be significantly different to that observed in Cas6 proteins, because the active sites of the two families are in different locations (Figure 4). In Cas5c, RNA is expected to bind to the helical face of the protein, which in all structures is positively charged, particularly adjacent to the active site [14,30]. Both domains of Cas5c are implicated in binding the substrate, including the β-sheet encompassing the putative β_2′_–β_3′_ hairpin [14,30]. However, neither the β_2_–β_3_ nor the β_2′_–β_3′_ hairpin can function by inserting at the base of the RNA hairpin, as this would place the scissile phosphate too far away from the active site. A complex structure of Cas5c and substrate is required to determine the exact mode of binding.

The method of RNA binding for Cas5c and Cas6 differs from typical RRMs, which contain the same ferredoxin-like fold as RAMPs. Typical RRMs possess two conserved sequence motifs located in β_1_ and β_3_ (termed RNP2 and RNP1 respectively) that are not present in RAMPs (Supplementary Figure S2 at http://www.biochemj.org/bj/453/bj4530155add.htm) [57,58]. These motifs allow RRMs to bind ssRNA or ssDNA across the face of the β-sheet [59,60], although not hairpin or dsRNA (double-stranded RNA), whereas RAMPs bind ssRNA or hairpin RNA through diverse modes of binding.

The active sites appear to have evolved independently for Cas6 and Cas5c, and even within the Cas6 family there is no universally conserved catalytic mechanism. Given that the catalytic rate constants of these enzymes, at 1–5 min^−1^ [37,40], are of the same order as those observed for catalytic RNA [61], these enzymes may be more constrained by the need to recognize pre-crRNA specifically than by a requirement to turn over rapidly.

## THE PROTEINS OF THE INTERFERENCE COMPLEXES

Atomic level detail structures are now available for a number of individual proteins that are involved in interference. In addition, EM structures have been solved for a number of the interference complexes (Figure 2). The highest resolution structures available are those of the *Escherichia coli e*Cascade in complex with crRNA and with a crRNA/protospacer RNA duplex at resolutions of 8 and 9 Å (1 Å=0.1 nm) respectively [39]. Lower resolution images and structures are also available for the *B. halodurans c*Cascade [14], *Ps. aeruginosa f*Cascade [15] and *S. solfataricus* CMR complex [20] as well as the core complex of *S. solfataricus a*Cascade [13]. Although the overall complex topologies can be discerned, the resolution of these structures has precluded reliable placement of individual proteins within the complex.

### Cas7, the backbone of the type I complex

The structural backbone of Cascade is composed of multiple monomers of Cas7 [13,14,39]. In *e*Cascade, Cas7 assembles into a helical hexameric structure with crRNA binding in a groove formed along the outer face of the oligomer [39]. This helical arrangement is conserved in the core complex of the *S. solfataricus a*Cascade, although this complex of Cas5 and Cas7 forms oligomers of variable length [13]. It is possible that further factors are needed to produce a complex of defined length or perhaps *a*Cascade exhibits greater structural plasticity than *e*Cascade. A similar helical arrangement to *e*Cascade was observed in EM images of *c*Cascade [14], and, although it was not possible to unambiguously define the quaternary structure of the complex, it is probable that the six Cas7 subunits of the complex form the same backbone. *f*Cascade contains six Csy3 subunits with a similar twisted topology to both *c*Cascade and *e*Cascade [15]. This, combined with secondary structure predictions and MS fragmentation analysis, has recently led to the hypothesis that Csy3 actually belongs in an expanded Cas7 family [56,62]. Similar structure predictions place Csc2 of *d*Cascade in the Cas7 family [56], suggesting that the Cas7 helical backbone is a conserved and perhaps characteristic feature of all Cascade complexes.

**Figure 5:**
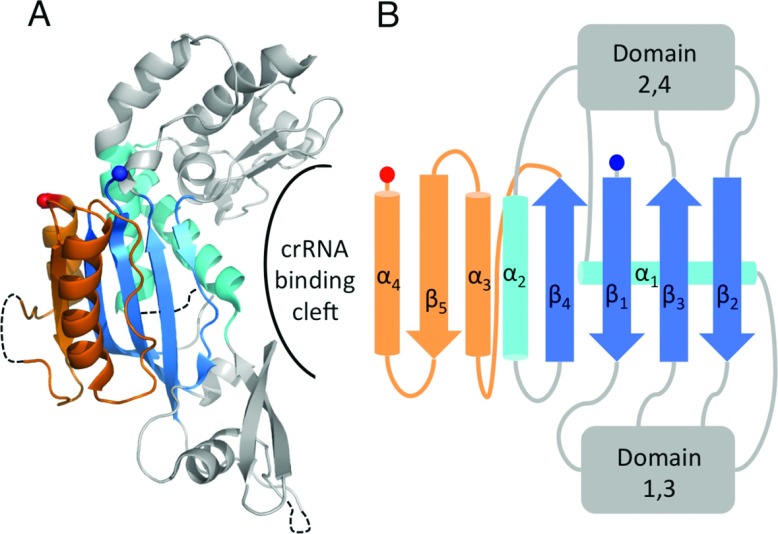
The structure of Cas7, the core subunit of Cascade (**A**) The structure of SsoCas7 (PDB code 3PS0) where the central RAMP domain is extended by an αβα motif (orange) and flanked by two unique domains (grey). The proposed crRNA-binding cleft located across the face of the β-sheet is indicated. (**B**) Topology diagram of SsoCas7 showing the connectivity of the RAMP fold relative to the other domains.

The structure of Cas7 from one of the *S. solfataricus a*Cascade complexes [13] (termed SsoCas7) contains a central RAMP fold modified with an additional αβα motif located immediately after β_4_ (Figure 5A). This motif adds a fifth strand to the β-sheet (β_5_β_4_β_1_β_3_β_2_) with the two helices on either side of β_5_. The loop between α_2_ and β_4_ is disordered in the structure and is not glycine-rich, a conserved feature of the Cas7 family [56]. Significant insertions are located between each of the four β-strands; these form two distinct regions above and below the β-sheet to form a crescent-shaped molecule (Figure 5B). Residues located in the cleft of SsoCas7 have been implicated in binding crRNA [13]. The structure of *e*Cascade shows that the *E. coli* Cas7 adopts a similar topology to SsoCas7 and that the cleft forms the extended groove along the helical assembly of Cas7 [39]. Given the likely ubiquitous nature of the Cas7 backbone, it is probable that all Cascade complexes bind crRNA in the same manner.

### Non-catalytic variants of Cas5

Although Cas5c possesses catalytic activity, the other members of the Cas5 family are non-catalytic and are limited to structural roles. In both *a*Cascade and *e*Cascade, Cas5 interacts stably with Cas7 [13,39]. Cas5e also interacts with Cse1 and Cse2 in *e*Cascade and appears to help stabilize the protospacer-bound conformation of the complex [39]. *c*Cascade contains two copies of Cas5c, which appear to occupy the positions of Cas5 and Cas6e in *e*Cascade [14,39]. Cas5c from *Streptococcus pyogenes* and *X. oryzae* bind dsDNA, which could be mimicking target dsDNA or the heteroduplex of the interference R-loop [52]. Therefore Cas5c seems to be able to function as both a catalytic Cas6 equivalent and a structural Cas5 equivalent.

Of the Cascade complexes, only *d*Cascade and *f*Cascade do not contain Cas5 [12]. On the basis of secondary structure predictions, Makarova et al. [56] predicted that Csc1 (I-D) and Csy2 (I-F) belong to the Cas5 family. EM images and the small-angle X-ray scattering (SAXS) structure of *f*Cascade place Csy2 in a similar position to the structural Cas5s of *c*Cascade and *e*Cascade [14,15,39]. However, the fragmentation patterns of *e*Cascade and *f*Cascade suggest that Csy2 does not interact with Csy3 (probable Cas7 equivalent) in the same manner as Cas5 and Cas7 from *e*Cascade, leading van Duijn et al. [62] to conclude that *f*Cascade does not contain a Cas5 equivalent. Further data are required to settle the relationships between the complexes.

### The small subunits of the interference complexes

**Figure 6:**
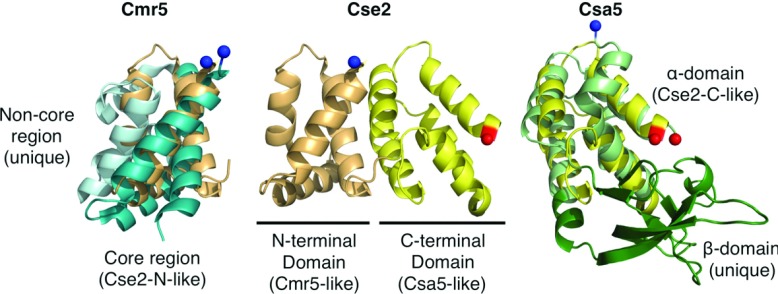
The small subunits of interference complexes Comparison of *T. thermophilus* Cmr5 (PDB code 2ZOP, left), *T. thermophilus* Cse2 (PDB code 2ZCA, middle) and *S. solfataricus* Csa5 (PDB code 3ZC4, right). The N-terminal domain of Cse2 (light orange) is superimposed on Cmr5 (blue) and the C-terminal domain of Cse2 (yellow) is superimposed on Csa5 (green).

Several of the interference complexes contain so-called ‘small’ subunits, which are typically <200 residues. These proteins are Csa5 (I-A), Cse2 (I-E), Csm2 (III-A) and Cmr5 (III-B) and it has been hypothesized that these proteins belong to a single family (Cas11) [56]. Analysis of the structures of Csa5 [63], Cse2 [64,65] and Cmr5 [66] (PDB codes 2OEB and 4GKF) shows that, although structural homology can be detected, the evolutionary links between the proteins are complex. Cse2 contains N- and C-terminal domains that consist of four and five α-helices respectively. The N-terminal domain is homologous with the core structure of Cmr5, whereas the C-terminal domain is homologous with one of the domains of Csa5 (Figure 6). Csa5 consists of an α-helical domain (homologous with the Cse2 C-terminal domain) and a β-sheet domain that is not homologous with Cse2 or Cmr5. In fact, this domain is very poorly conserved across the Csa5 family and is likely to vary significantly between homologues.

Possible evolutionary scenarios for the homology include fusion of *csa5* and *cmr5* genes to form *cse2* or the evolution of the three proteins from a single *cse2*-like gene with domain loss to form Csa5 and Cmr5 [63]. Csm2, the remaining small subunit for which there is no structure available, may be critical for determining the likely scenario, although it is certainly possible that Csm2 may not possess any homology with the other small subunits. Makarova et al. [56] suggested that the Cas8 C-terminal domain, which is predicted to be helical, might be homologous with the small subunits, although no experimental structure exists to confirm this.

The Cse2 dimer is an integral part of *e*Cascade [39] and is responsible for stabilizing the R-loop, increasing the affinity of *e*Cascade for dsDNA approximately 10-fold [67]. Cse2 alone binds non-specifically to dsDNA and ssRNA [65]. Conversely, the *S. solfataricus* Csa5 does not stably interact with Cas5/Cas7 in the presence of crRNA or with nucleic acids alone [63]. Cmr5, in contrast with both Csa5 and Cse2, appears to be non-essential to the function of the CMR complex [11]. Thus we conclude that the similarity of the small subunits is structural rather than functional.

### The large subunits of the interference complexes

Similarly to the small subunits, each of the type I and III interference complexes contains a ‘large’ (>500 residues) subunit: Cas8 (I-A, I-B, I-C), Cse1 (I-E), Csy1 (I-F) and Cas10 (I-D, III-A and III-B). Cas10 was originally predicted to be a polymerase (hence the name polymerase cassette for the III-B subtype) on the basis of sequence features typical of a palm domain commonly found in polymerases and cyclases [44]. Subsequently it was proposed that all of the large subunits were homologous and part of a Cas10 superfamily [56]. However, recent structures of a type III-B Cas10 [68,69], denoted Cas10b, show that, although the prediction of the palm domain was correct (albeit more akin to cyclases), no significant structural homology exists with Cse1 [70,71] (PDB codes 4H3T and 4EJ3). This argues against a single common ancestor for all of the large subunits.

### Cas10, the large subunit of type III systems

**Figure 7:**
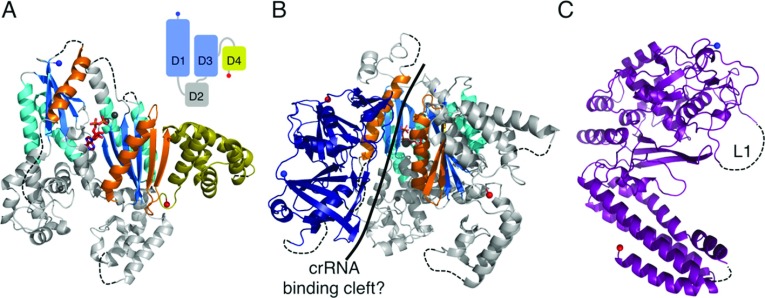
The large subunits of interference complexes (**A**) The structure of PfuCas10b^dHD^ (PDB code 3UNG) in complex with ADP (red sticks). The ferredoxin-like folds are coloured as for RAMPs and the additional adenylate cyclase elements are shown in orange. D4 is shown in yellow to highlight its homology with the small subunits. The three metal ions are shown as grey spheres. Inset: schematic diagram showing the relative positions of the four domains (D1–D4) with the cyclase-like domains in blue and the small subunit-like domain in yellow. (**B**) The structure of the Cas10b^dHD^–Cmr3 complex (PDB code 4H4K) with Cmr3 shown in navy blue and Cas10b^dHD^ as in (**A**). The putative crRNA-binding cleft is indicated with a solid black line. (**C**) The structure of Cse1 from *T. thermophilus* (PDB code 4AN8) with the disordered loop L1 indicated.

Cas10 is the defining protein of the type III system and consists of an N-terminal HD (histidine–aspartate) phosphohydrolase domain (for which there is no structure) and a C-terminal region (Cas10^dHD^) that contains the palm domain [56]. Cas10b^dHD^ from *P. furiosus* consists of two adenylate cyclase-like domains (denoted D1 and D3) and two α-helical domains (D2 and D4) (Figures 7A and 7B) [68,69]. D2 is not significantly homologous with known structures, but D4 is structurally homologous with Cmr5 and the N-terminal domain of Cse2, although sequence conservation is minimal and the biological implications of the homology are unclear. A typical adenylate cyclase domain consists of a ferredoxin-like fold with a C-terminal α_3_β_5_α_4_β_6_β_7_ modification, which creates a seven-stranded β-sheet with the two additional helices located on either side of the sheet [72]. D1 and D3 lack some of these key structural elements: D3 lacks α_4_ and β_6_, whereas D1 lacks every additional element bar α_3_. Individually, D1 and D3 are most similar to the type III adenylate cyclase from *Mycobacterium tuberculosis* [72]. However, these bacterial cyclases are typically homodimers, whereas D1 and D3 of Cas10b^dHD^ exist as a pseudoheterodimer more similar to the arrangement of mammalian cyclases [73]. The orientation between D1 and D3 is markedly different to that of typical cyclases which, combined with the loss of key structural and sequence features, is consistent with PfuCas10b^dHD^ lacking a cyclase-like catalytic activity, although D3 retains the ability to bind ADP [68].

In the CMR complex Cas10b interacts with Cmr3, an interaction observed in both *S. solfataricus* and *P. furiosus* [20,74]. The structure of the *P. furiosus* Cas10b^dHD^–Cmr3 complex shows that the two proteins form a heterodimer with the interface formed by D1 of Cas10b^dHD^ and one face of Cmr3 (see below) [74]. At the interface between the two proteins is a highly positively charged cleft ~50 Å in length, which is suggestive of a role in crRNA binding. The nucleotide bound by D3 in both the Cas10b^dHD^ and Cas10b^dHD^–Cmr3 structures lies at the centre of this cleft and so could be mimicking crRNA binding by the complex rather than substrate binding by the ‘cyclase’ domains of Cas10b^dHD^. This is consistent with the nucleotide binding in a different orientation to that observed in cyclases.

If the Cas10b–Cmr3 complex does bind to part of the crRNA, the remainder of the crRNA must be bound by other subunits of the CMR complex. Three subunits of the complex (Cmr1, Cmr4 and Cmr6) are RAMPs and thus are plausible candidates. Makarova et al. [56] have predicted Cmr4 and Cmr6 to be Cas7 homologues. However, EM structures of the CMR complex (which targets ssRNA and not dsDNA) show that it is more compact than Cascade and lacks a central helical structure [20].

### Cse1, the PAM (protospacer adjacent motif) sensor of *e*Cascade

The structures of Cse1 from *T. thermophilus* [70,71] (PDB code 4EJ3) and *Acidimicrobium ferrooxidans* (PDB code 4H3T) consist of an N-terminal mixed α/β domain with a novel fold and a C-terminal four-helix bundle (Figure 7C). In *e*Cascade, Cse1 is responsible for recognition of the PAM, a short (2–5 nt) conserved sequence located immediately next to the protospacer that is required for interference [75]. Cascade recognizes a PAM located 5′ to the protospacer [75] and, at least for *e*Cascade, PAM recognition uses the complementary strand [76]. Target dsDNA lacking a PAM is bound weakly by *e*Cascade [76,77] and is resistant to cleavage [78], consistent with the observation that mutations in the PAM can prevent interference [15,79].

The N-terminal domain of Cse1 contains a loop (L1, Figure 7C) that is disordered in all of the available crystal structures, but is critical for PAM recognition [70,71]. Analysis of the *e*Cascade structures led Mulepati et al. [70] and Sashital et al. [71] to suggest that L1 binds to the crRNA 5′-handle and PAM in the absence and presence of target DNA respectively. Cse1 is also critical for binding to negatively supercoiled dsDNA, both specifically to a protospacer and also non-specifically, a function that is dependent on the L1 loop [53,70,71]. Sashital et al. [71] have proposed that Cse1 scans dsDNA for PAM sequences and once in contact destabilizes the duplex to allow for target recognition, first through a 5′ seed sequence and then along the remainder of the target.

Other Cascade complexes lack Cse1 and must use a different protein for PAM sensing, although their identities have not been established. Cas8 and Csy1 are candidates as they dissociate easily from their respective complexes (similar to Cse1 and *e*Cascade) and EM images suggest that they are located in a similar position to Cse1 within their complexes [14,39,62].

### Cmr3, a type III-B Cas6-like protein

**Figure 8:**
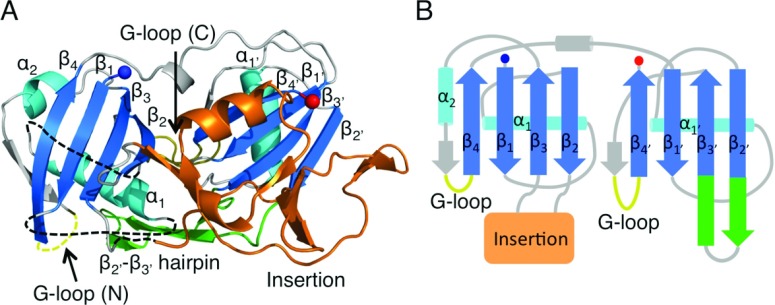
The structure of Cmr3 (**A**) The structure of PfuCmr3 (PDB code 4H4K) showing the RAMP elements and the structural insertion in the N-terminal domain (orange). (**B**) Topology diagram of PfuCmr3 highlighting the conserved RAMP features and the connectivity of the insertion domain.

Cmr3 is a RAMP protein of the CMR complex and the structure of PfuCmr3, available only in complex with Cas10b^dHD^, shows that it contains two RAMP domains arranged in a similar manner to Cas6 (compare Figure 8 with Figure 3B) [74]. The C-terminal domain contains two of the conserved features of Cas6: the β_2′_–β_3′_ hairpin and the glycine-rich loop, both of which adopt similar conformations to those seen in Cas6 proteins. The Cmr3 glycine-rich loop also exhibits a similar consensus sequence to that of Cas6 (XXXXXGϕG, where ϕ is an aromatic residue, X is any residue and the variable region contains at least one positively charged residue) (Supplementary Figure S3 at http://www.biochemj.org/bj/453/bj4530155add.htm). In the N-terminal domain, a β-strand located after α_2_ forms a β-hairpin with β_4_, as is also seen in the *Pyrococcus* and *Sulfolobus* Cas6 homologues [46–50], with the turn of the hairpin containing the two conserved glycine residues identified by Makarova et al. [56] as an N-terminal glycine-rich loop. The tip of this loop is disordered, but since it is only three residues in length it acts more as a turn rather than the extended loop seen in many RAMPs.

Cmr3 exhibits two significant deviations from Cas6. α_2′_ is replaced by a short β-strand located immediately prior to the C-terminal glycine-rich loop, similar to the β-strand located before the N-terminal glycine-rich loop. The second difference is the presence of a significant structural insertion located between β_2_ and β_3_ of the N-terminal domain. This insertion consists of two short helices and seven β-strands and packs against the C-terminal β-sheet. The insertion and the β_2′_–β_3′_ hairpin together form the interface with Cas10b^dHD^ and line the putative crRNA-binding cleft.

## THE INTERFERENCE NUCLEASES

During interference, invading nucleic acids detected by base pairing with crRNA are targeted for degradation by an interference nuclease. In type I systems this is the HD metal-dependent nuclease domain of Cas3, which is recruited to Cascade rather than being an integral component [76]. Type II systems use Cas9 as the sole interference protein with the HNH-like and RuvC-like nuclease domains cleaving the complementary and non-complementary strands of the R-loop respectively [16,80]. The interference nucleases of the type III systems are unknown. The nuclease is within the CMR complex, but Cas10b and Cmr5 have been discounted, as has the *Sulfolobales*-specific protein Cmr7 [11,20,68].

### Cas3, the interference nuclease of type I systems

Cas3 is the defining protein of the type I system and consists of an N-terminal HD nuclease domain and a C-terminal superfamily II DExD/H-box helicase domain [12,44,81]. In some systems the two domains are expressed as separate proteins (Cas3″ and Cas3′ respectively); other variations are also known, such as domain fusion to other Cas proteins (for example, Cas3–Cas2 in the I-F subtype and Cas3–Cse1 in some I-E systems) and inversion of the domain order (Figure 2) [12,44,76]. Cas3 is recruited by Cascade after R-loop formation where it catalyses the unwinding and degradation of the invading DNA [76,78].

**Figure 9:**
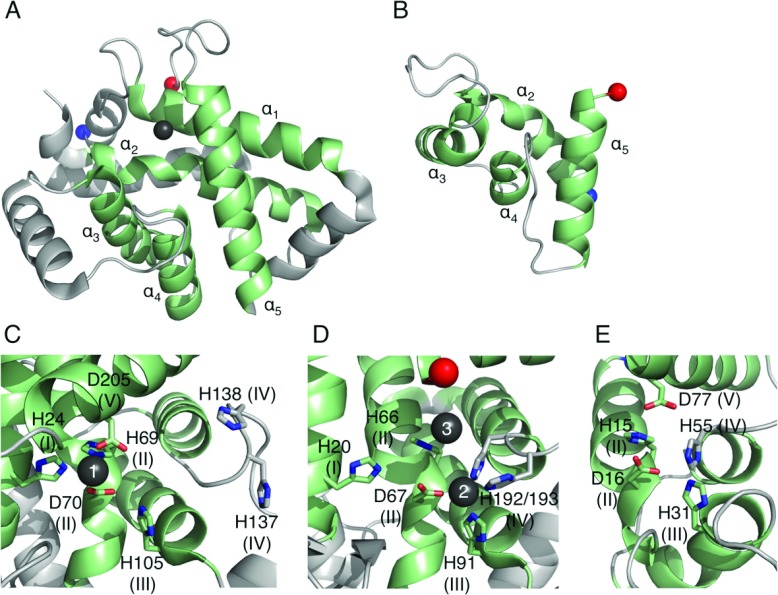
The structures of Cas protein HD domains (**A**) The structure of TtCas3^HD^ (PDB code 3SKD) with the conserved HD superfamily helices in green and numbered. The Ni^2+^ ion is shown as a dark grey sphere. Residues 222–260 are not shown as they are predicted to belong to the helicase domain. (**B**) A homology model of the HD domain of Cas10a from *S. thermophilus* created using PHYRE2 and consisting of residues 4–79. The four HD domain helices are coloured in green and labelled. (**C**–**E**) Views of the active sites of (**C**) TtCas3^HD^, (**D**) MjaCas3″ (PDB code 3S4L) and (**E**) SthCas10a^HD^. The HD superfamily motifs are shown as sticks with motif numbers in parentheses and the metal ions as grey spheres with site numbers in white.

Cas3 proteins contain all five HD superfamily sequence motifs (H-HD-H-H-D) and the structures of TtCas3^HD^ (HD domain of TtCas3) and MjaCas3″ (*Methanocaldococcus jannaschii* Cas3″) revealed eight conserved helices, five of which are characteristic of the HD superfamily (Figure 9A) [82,83]. In the TtCas3^HD^ structure a single Ni^2+^ ion is bound by motifs I, II and V (site 1), whereas site 2 (a binding site formed by motifs II, III and IV) remains unoccupied (Figure 9C). Metal binding at site 2 has been observed in a number of HD domains (for example, see PDB codes 2OGI, 2O08, 2PQ7, 3CCG and 3HC1) and its absence in the TtCas3^HD^ structure is likely to be a crystal artefact. The MjaCas3″ structure shows a Ca^2+^ ion bound at site 2 as well as a second ion bound by the histidine of motif II (site 3) (Figure 9D). However, the binding at site 3 and the lack of binding at site 1 are likely to be artefacts resulting from the protein engineering required for crystallization.

Characterization of type I-E Cas3 nuclease domains from *T. thermophilus*, *Streptococcus thermophilus*, and *E. coli* and the type I-A Cas3″ proteins from *M. jannaschii* and *P. furiosus* showed that they are all metal-dependent nucleases specific for ssDNA, although the Cas3″ proteins also cleave ssRNA *in vitro* [82–84]. These proteins are both endo- and exo-nucleases, with the latter activity proceeding in the 3′→5′ direction. MjaCas3″, SthCas3 (*Streptococcus thermophilus* Cas3) and EcoCas3 (*E. coli* Cas3) cleave R-loops, the biological substrate of Cas3 and MjaCas3″ and SthCas3 have been shown to target the non-complementary ssDNA strand specifically [76,78,82]. Structural data is not available for the helicase domain of Cas3, but the type I-E helicase domains of SthCas3 and EcoCas3 catalyse the 3′→5′ Mg^2+^- and ATP-dependent unwinding of dsDNA and DNA/RNA duplexes [84,85]. Nicking of the non-complementary strand by the HD domain followed by the unwinding of the DNA duplex by the helicase domain would allow for progressive degradation of the non-complementary strand (Supplementary Figure S4 at http://www.biochemj.org/bj/453/bj4530155add.htm). The complementary strand is also targeted by Cas3 [78] and would occur after dissociation of DNA from the R-loop.

### The HD domains of Cas10 proteins

Cas10 proteins contain N-terminal HD domains that are highly divergent from typical HD domains, being both shorter than classical HD proteins and lacking characteristic motifs (Supplementary Figure S5 at http://www.biochemj.org/bj/453/bj4530155add.htm) [86]. A homology model of Cas10a from *S. thermophilus* built using PHYRE2 [87] shows that motifs II, III and IV could co-ordinate a metal ion in a similar way to that of site 2 of Cas3 (Figures 9B and 9E). Therefore this domain could also be catalytically active and might potentially act as the interference nuclease of the CSM complex, although so far experimental confirmation is lacking. In contrast, Cas10b only contains motif II and so is unlikely to be an active nuclease, consistent with the observation that the Cas10b HD domain is not necessary for interference by the CMR complex [68], perhaps unsurprising since this complex targets RNA.

## CONCLUDING REMARKS

The structural biology of the CRISPR system provides a wealth of information on the evolution and mechanisms of the proteins involved. It has revealed the underlying relationships between highly divergent proteins that are difficult or impossible to detect using bioinformatic approaches (however heroic) alone. The RAMP (or RAMP-like) domains, present in the Cas2, Cas5, Cas6, Cas7, Cas10 and Cmr3 families, are the leitmotif of the system, providing RNA-binding and -cleavage functionalities that are central to the process. The backbone of all type I complexes is likely to be a helical arrangement of Cas7, and a similar arrangement of Cas7-like RAMP subunits may be found in the CSM complex, given that it, too, targets dsDNA. Key challenges for crystallography include the structure of the Cas9 protein of type II systems, which has so far evaded attempts to place it in a wider context. Structures of the large and small subunits of the various type I and type III-A complexes are expected to clarify the relationships between the different families, and we can look forward to some simplification of the overall picture as these relationships become apparent. Finally, atomic level structural information on the ~400 kDa CRISPR interference complexes remains a grand challenge in molecular biology, one that has been taken up enthusiastically by the structural biology community.

## Online data

Supplementary data
