# Supplementary material for: CRISPR interference: a structural perspective
Source: Biochem J. 2013 Jun 28;453(Pt 2):155–66. doi: 10.1042/BJ20130316 (PMC3727216; doi:10.1042/BJ20130316)
Supplement: Supplementary data [file bj4530155add.pdf]

BJ Biomolecules

Biochem. J. (2013) **453**, 155–166 (Printed in Great Britain) doi:10.1042/BJ20130316

multimedia online data

SUPPLEMENTARY ONLINE DATA
CRISPR interference: a structural perspective

Judith REEKS, James H. NAISMITH[1] and Malcolm F. WHITE[1]

Biomedical Sciences Research Complex, University of St Andrews, St Andrews, Fife KY16 9ST, U.K.

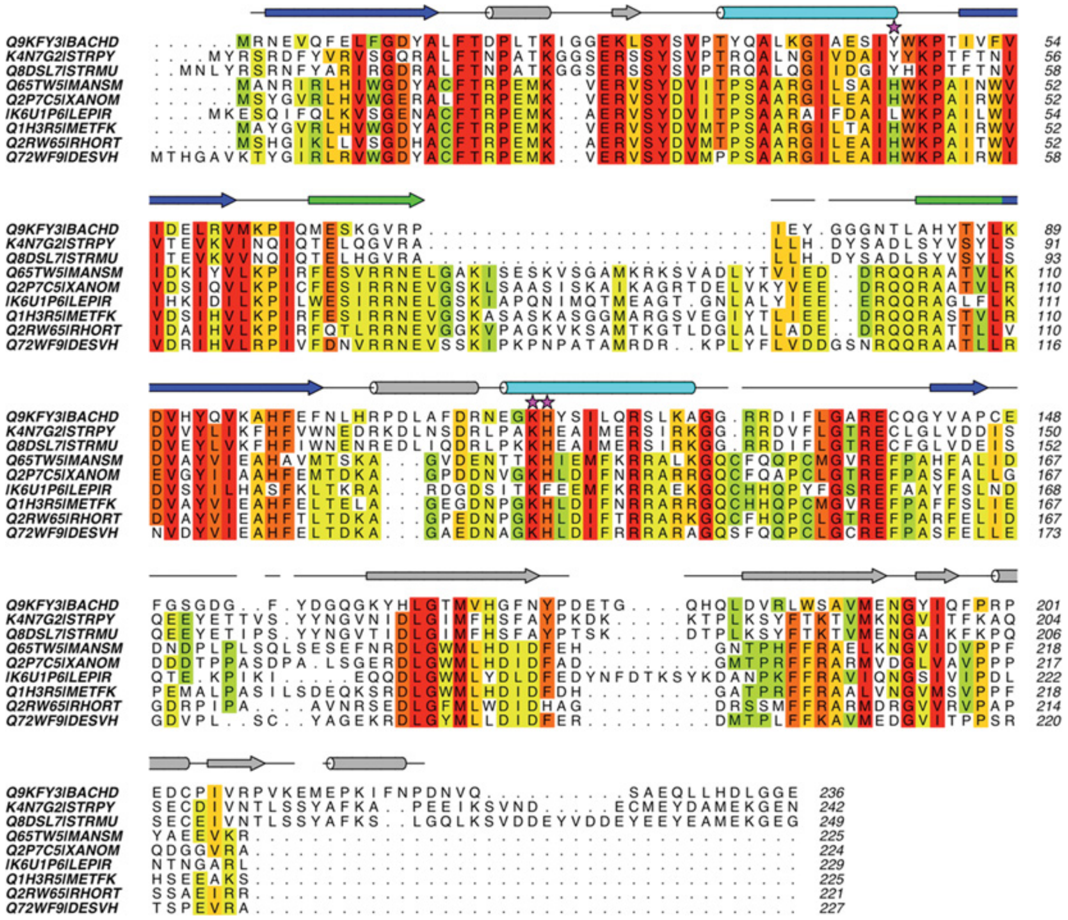

Figure S1 Sequence alignments of Cas5c proteins

Sequence similarity is shaded from red (highest) to green (lowest). The secondary structure elements of BhCas5c are shown above the alignments and are coloured according to Figure 3 of the main text. Gaps in the elements represent disordered residues. The catalytic residues of BhCas5c are indicated by magenta stars.

[1] Correspondence may be addressed to either of these authors (email naismith@st-and.ac.uk or mfw2@st-and.ac.uk).

© 2013 The Author(s)
The author(s) has paid for this article to be freely available under the terms of the Creative Commons Attribution Licence (CC-BY) (http://creativecommons.org/licenses/by/3.0/) which permits unrestricted use, distribution and reproduction in any medium, provided the original work is properly cited.

Biochemical Journal www.biochemj.org

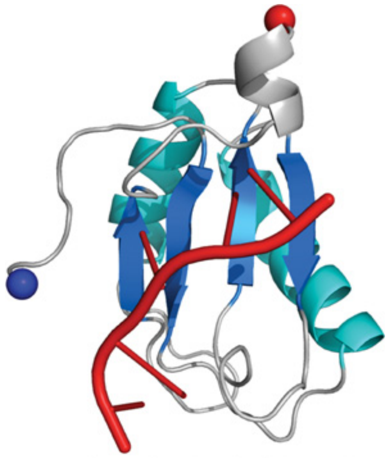

Figure S2 The structure of U1A spliceosomal protein, a typical RRM protein, in complex with RNA (red) (PDB code 1URN)

For clarity, the unbound RNA hairpin is not shown. The two RRM RNA-binding consensus sequences are shown beneath the structure. The RPM domain is coloured in the same manner as the RAMP domain in the main text.

© 2013 The Author(s)
The author(s) has paid for this article to be freely available under the terms of the Creative Commons Attribution Licence (CC-BY) (http://creativecommons.org/licenses/by/3.0/) which permits unrestricted use, distribution and reproduction in any medium, provided the original work is properly cited.

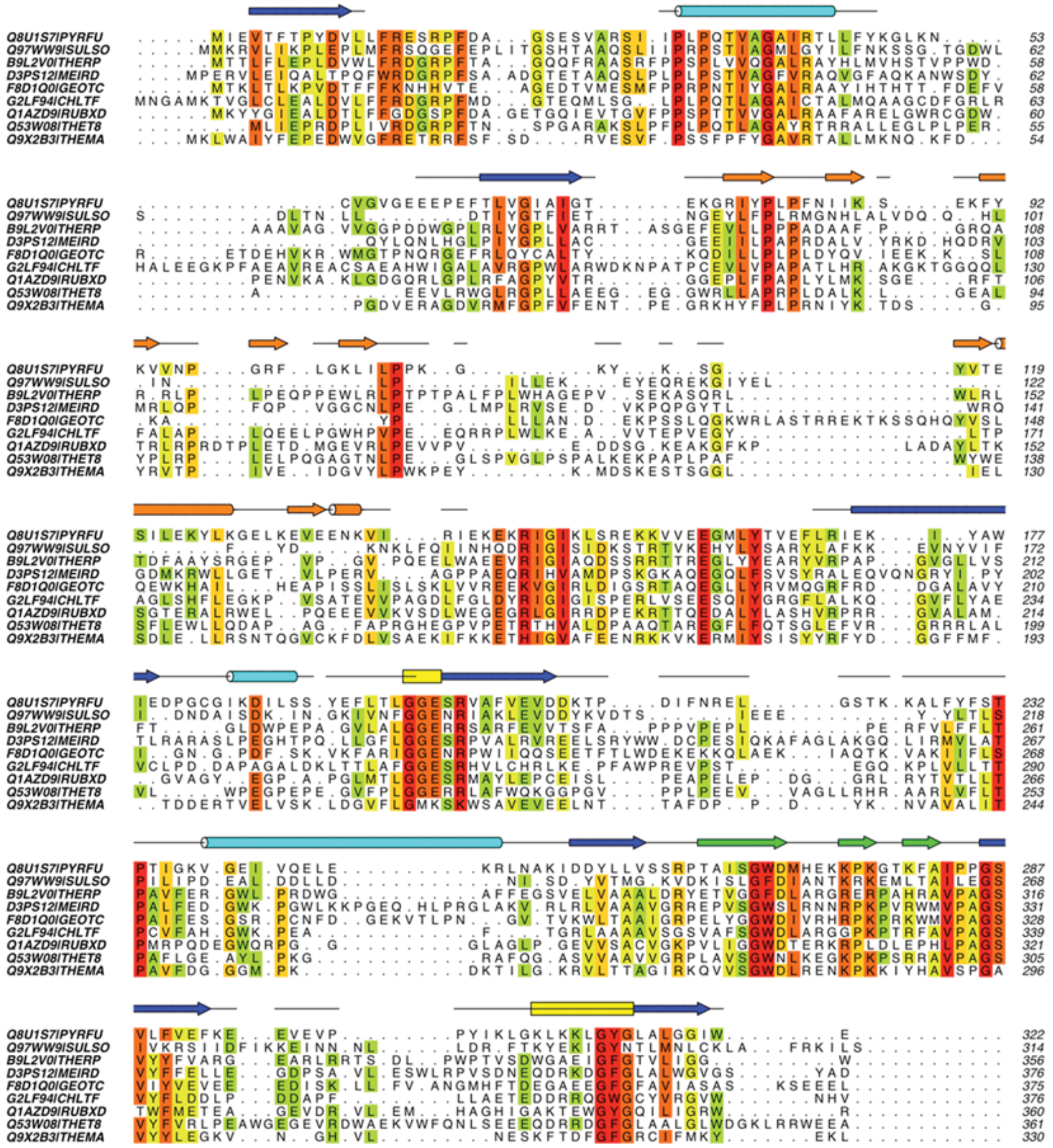

Figure S3 Sequence alignment of Cmr3 proteins

Sequence similarity is shaded from red (highest) to green (lowest). The secondary structure elements from PfuCmr3 are shown above and coloured according to Figure 8 of the main text.

© 2013 The Author(s)
The author(s) has paid for this article to be freely available under the terms of the Creative Commons Attribution Licence (CC-BY) (http://creativecommons.org/licenses/by/3.0/) which permits unrestricted use, distribution and reproduction in any medium, provided the original work is properly cited.

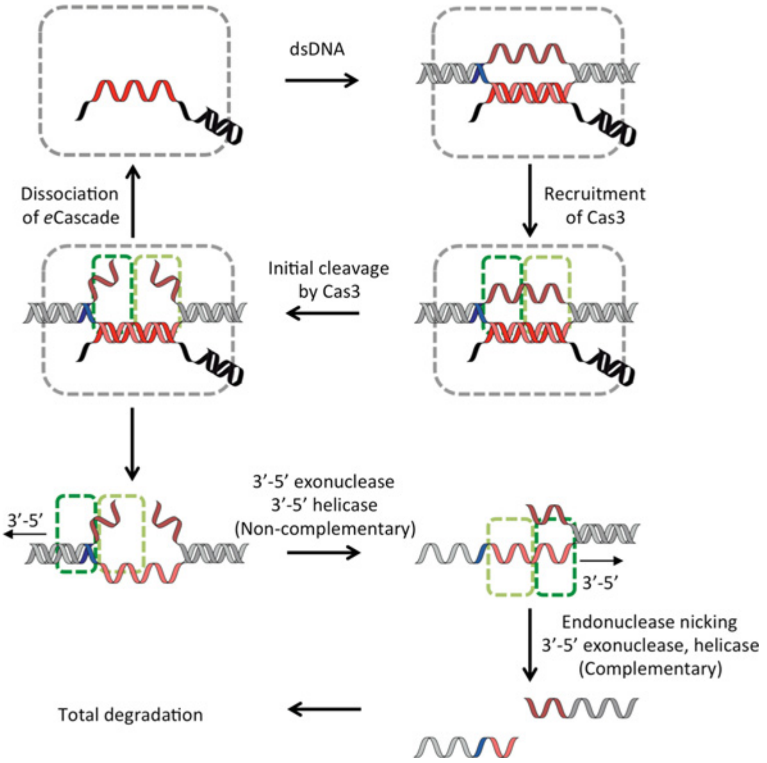

Figure S4 Schematic diagram of dsDNA degradation by Cas3 in the type I-E system

Repeats are shown in black, protospacers and spacers in red, PAMs in blue and DNA in grey. The Cas3 HD domain is represented by a light green dotted line, the Cas3 helicase domain in dark green and *e*Cascade by a grey line.

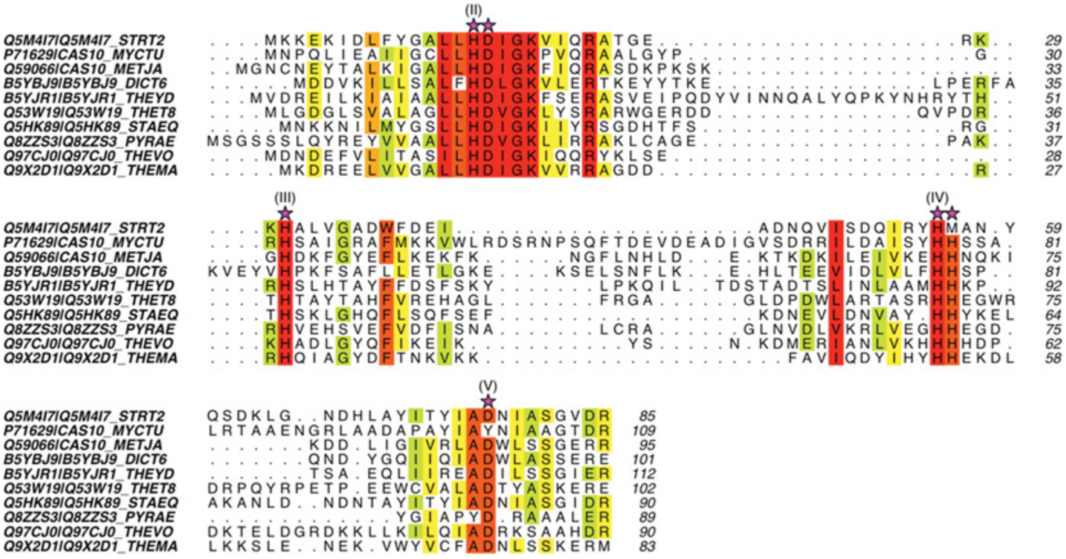

Figure S5 Sequence alignments of the HD domains of Cas10a

The putative HD superfamily sequence motifs are highlighted with magenta stars and the motif number is indicated.

© 2013 The Author(s)
The author(s) has paid for this article to be freely available under the terms of the Creative Commons Attribution Licence (CC-BY) (http://creativecommons.org/licenses/by/3.0/) which permits unrestricted use, distribution and reproduction in any medium, provided the original work is properly cited.

Table S1 Details of all of the crystal structures of Cas proteins available at the time of writing

Protein	Organism	PDB code(s)	Notes
Cas1	*Aquifex aeolicus*	2YZS	
Cas1	*Escherichia coli*	3NKD, 3NKE	
Cas1	*Pseudomonas aeruginosa*	3GOD	
Cas1	*Pyrococcus horikoshii*	3PV9	
Cas1	*Thermotoga maritima*	3LFX	
Cas2	*Bacillus halodurans*	4ES1, 4ES2, 4ES3	
Cas2	*Desulfovibrio vulgaris*	3OQ2	
Cas2	*Pyrococcus furiosus*	2I0X	
Cas2	*S. solfataricus*	2IVY, 2I8E, 3EXC	Two paralogues
Cas2	*Thermus thermophilus*	1ZPW	
Cas3	*T. thermophilus*	3SK9, 3SKD	HD domain only
Cas3′′	*Methanocaldococcus jannaschii*	3S4L	
Cas4	*S. solfataricus*	4IC1	
Cas5c	*Mannheimia succiniciproducens*	3KG4	
Cas5c	*Bacillus halodurans*	4F3M	
Cas5c	*Streptococcus pyogenes*	3VZH	
Cas5c	*Xanthomonas oryzae*	3VZI	
Cas6	*E. coli*	4DZD	
Cas6	*P. furiosus*	3I4H, 3PKM, 3UFC	Two paralogues
Cas6	*P. horikoshii*	3QJJ, 3QJL, 3QJP	
Cas6	*S. solfataricus*	3ZFV, 4ILL, 4ILM, 4ILR	Two paralogues
Cas6e	*T. thermophilus*	1WJ9, 2Y8W, 2Y8Y, 2Y9H, 3QRP, 3QRQ, 3QRR	
Cas6f	*Ps. aeruginosa*	2XLI, 2XLJ, 2XLK, 4AL5, 4AL6, 4AL7	
Cas7	*S. solfataricus*	3PS0	
Cas10b^{dHD}	*P. furiosus*	3UNG, 3UR3, 4DOZ, 4H4K	Lacking HD domain, also in complex with Cmr3
Csm6	*S. solfataricus*	3QYF	
Csn2	*Enterococcus faecalis*	3S5U	
Csn2	*Streptococcus agalactiae*	3QHQ	
Csn2	*S. pyogenes*	3TOC, 3V7F	
Csn2	*Streptococcus thermophilus*	3ZTH	
Cmr3	*P. furiosus*	4H4K	In complex with Cas10b^{dHD}
Cmr5	*Archaeglobus fulgidus*	2OEB	
Cmr5	*P. furiosus*	4GKF	
Cmr5	*T. thermophilus*	2ZOP	
Cmr7	*S. solfataricus*	2XVO, 2X5Q	Two paralogues, *Sulfolobales*-specific
Csa3	*S. solfataricus*	2WTE	
Csa5	*S. solfataricus*	3ZC4	
Cse1	*Acidimicrobium ferrooxidans*	4H3T	
Cse1	*T. thermophilus*	4AN8, 4F3E, 4EJ3	
Cse2	*Thermobifida fusca*	4H79	
Cse2	*T. thermophilus*	2ZCA, 4H7A	
Csn2	*Streptococcus thermophilus*	3ZTH	
Csx1	*P. furiosus*	4EOG	
Csx1	*S. solfataricus*	2I71	

Received 4 March 2013/9 April 2013; accepted 17 April 2013
Published on the Internet 28 June 2013, doi:10.1042/BJ20130316

© 2013 The Author(s)
The author(s) has paid for this article to be freely available under the terms of the Creative Commons Attribution Licence (CC-BY) (http://creativecommons.org/licenses/by/3.0/) which permits unrestricted use, distribution and reproduction in any medium, provided the original work is properly cited.
